# Microencapsulation of *Piscirickettsia salmonis* Antigens for Fish Oral Immunization: Optimization and Stability Studies

**DOI:** 10.3390/polym14235115

**Published:** 2022-11-24

**Authors:** Daniela Sotomayor-Gerding, José Miguel Troncoso, Katherine Díaz-Riquelme, Karin Mariana Torres-Obreque, Juan Cumilaf, Alejandro J. Yañez, Mónica Rubilar

**Affiliations:** 1 Programa de Doctorado en Ciencias de Recursos Naturales, Universidad de La Frontera, Avenida Francisco Salazar 01145, Box 54-D, Temuco 4811230, Chile; 2 Cargill Innovation Center, Camino a Pargua km 57, Colaco km 5, Calbuco 5570130, Chile; 3 Doctorado en Acuicultura, Programa Cooperativo Universidad de Chile-Universidad Católica del Norte-Universidad Católica de Valparaíso, Macul 7830490, Chile; 4 Department of Biochemical and Pharmaceutical Technology, School of Pharmaceutical Sciences, University of São Paulo, São Paulo 05508-000, SP, Brazil; 5 Doctorate in Engineering Sciences with Specialization in Bioprocesses, Universidad de La Frontera, Avenida Francisco Salazar 01145, Box 54-D, Temuco 4811230, Chile; 6 Interdisciplinary Center for Aquaculture Research (INCAR), Concepción 407007, Chile; 7 Facultad de Ciencias, Universidad Austral de Chile, Valdivia 5090000, Chile; 8 Department of Chemical Engineering, Faculty of Engineering and Sciences, Universidad de La Frontera, Avenida Francisco Salazar 01145, Box 54-D, Temuco 4811230, Chile; 9 Scientific and Technological Bioresource Nucleus, BIOREN, Universidad de La Frontera, Avenida Francisco Salazar 01145, Box 54-D, Temuco 4811230, Chile

**Keywords:** *Piscirickettsia salmonis*, oral vaccine, alginate, fish feed, vacuum coating, particle size, encapsulation efficiency, antigen, stability, Micro-CT-scanning

## Abstract

The development of fish oral vaccines is of great interest to the aquaculture industry due to the possibility of rapid vaccination of a large number of animals at reduced cost. In a previous study, we evaluated the effect of alginate-encapsulated *Piscirickettsia salmonis* antigens (AEPSA) incorporated in feed, effectively enhancing the immune response in Atlantic salmon (*Salmo salar*). In this study, we seek to characterize AEPSA produced by ionic gelation using an aerodynamically assisted jetting (AAJ) system, to optimize microencapsulation efficiency (EE%), to assess microparticle stability against environmental (pH, salinity and temperature) and gastrointestinal conditions, and to evaluate microparticle incorporation in fish feed pellets through micro-CT-scanning. The AAJ system was effective in obtaining small microparticles (d < 20 μm) with a high EE% (97.92%). Environmental conditions (pH, salinity and temperature) generated instability in the microparticles, triggering protein release. 62.42% of the protein content was delivered at the intestinal level after in vitro digestion. Finally, micro-CT-scanning images confirmed microparticle incorporation in fish feed pellets. In conclusion, the AAJ system is effective at encapsulating *P. salmonis* antigens in alginate with a high EE% and a size small enough to be incorporated in fish feed and produce an oral vaccine.

## 1. Introduction

The development of fish oral vaccines is of great interest to the aquaculture industry, considering its advantages over injectable vaccines, which include easy administration, improved safety, significant stress reduction for the fish and the possibility of rapidly vaccinating a large quantity of fish with reduced costs [1,2]. Oral vaccines for fish have been evaluated since 1942 [3]; different formulations have been tested for the control of pathogens in several species [2], including *Aeromonas hydrophila* in catla, rohu and common carp [4], *Edwarsiella tarda* in olive flounder [5], *Streptococcus agalactiae* in Nile tilapia [6], *Vibrio anguilarum* in Atlantic halibut and turbot [7,8], among others. Particularly for salmonids, oral vaccines have been tested for the control of viral hemorrhagic septicemia virus (VHSV) [1], infectious hematopoietic necrosis virus (IHNV) [9], infectious salmon anemia virus (ISAV) [10,11], infectious pancreatic necrosis virus (IPNV) [12,13,14] and salmon rickettsial septicemia (SRS) [15,16,17].

Improving current oral vaccination strategies is an important issue in fish vaccine development. Studies comparing the immune response and efficacy of vaccines after oral or anal administration have shown the need to protect the antigen [18,19] from degradation in the digestive system of the fish [20]. In addition, there is a need to prevent the interaction of the antigen with other feed components that could prevent its arrival in the hindgut where its absorption will occur [21].

Microencapsulation of immunological agents inside polymeric devices constitutes an attainable strategy for effective oral antigen delivery to fish [22]. Previous studies have evaluated the use of polymers such as chitosan, alginate or poly D, L-lactic-co-glycolic acid (PLGA) and other types of encapsulating agents such as liposomes or the patented technology MicroMatrix^TM^ [2]. Among the evaluated polymers, alginate microparticles have the advantage of an excellent biocompatibility within host tissues [23]; in addition, studies have shown their ability to remain stable at an acidic pH, protecting the bioactives from the harsh environment of gastric digestion and allowing bioactive release in the intestinal phase [24,25]. After diffusion through the gut mucus layer and upon reaching the enterocyte surface, the antigens might be actively taken up by antigen-sampling cells [2], initiating the effect of the oral vaccine. These properties make alginate particularly suitable for the oral delivery of antigens to the fish. Furthermore, alginate has been used in several studies for the encapsulation of antigens from bacterial (*Vibrio harveyi*, *Flavobacterium psychrophilum*, *Lactococcus garviae*) and viral (IHNV and IPNV) pathogens that affect salmonids with effective results [2]. In our previous study, the effects of alginate-encapsulated *Piscirickettsia salmonis* antigens incorporated in the feed were evaluated, without affecting the acceptability of the feed or the fish growth and effectively enhancing the immune response of the fish [17].

Although the use of alginate in microencapsulation is well documented [23,26], its use in the development of oral vaccines is still under study [27]. The success of a developed encapsulation system depends mainly on the encapsulation technique used, the optimization of the process conditions and the polymers used as encapsulating agents. All these parameters can affect both the process and the encapsulation efficiency [28]. In this study, we used the aerodynamically assisted jetting (AAJ) system for microparticle dispersion, where the primary variables that could affect the process are as follows: flow rate of alginate solution, alginate concentration, distance between spray head and cross-linking solution, and air pressure [29]. This technology is capable of producing small-sized capsules [30], which is a very important characteristic, since these microparticles must be incorporated into the pores of the pellet where most of the pores have a size close to 100 microns [29]. Additionally, this technique has previously demonstrated its effectiveness for the encapsulation of IPNV antigens for the development of an oral vaccine [31].

On the other hand, the evaluation of conditions affecting the microparticle stability and triggering the release of the bioactive is indispensable to estimate the dose of bioactive that remains available for absorption after being supplied, consumed and digested by the fish [32]. Environmental factors (pH, ionic strength and temperature) encountered during food processing have been evaluated and found to affect the stability of microencapsulation systems considerably [33,34].

Finally, to develop an oral vaccine, microparticle incorporation in fish feed pellets is essential. We used the vacuum coating technology to incorporate microparticles into the pores of the feed matrix and found slight evidence of microparticle incorporation through scanning electron microscopy in a previous study [17]; however, evaluation through high resolution micro-computed tomography could provide us with a better visualization of the microparticles and their distribution within the pellet [35].

Consequently, the main objectives of this study were as follows: (i) to optimize antigen microencapsulation conditions to improve encapsulation efficiency, (ii) to evaluate the stability of the alginate-encapsulated SRS antigens and (iii) to evaluate microparticle incorporation in fish feed pellets through micro-CT scanning.

## 2. Materials and Methods

### 2.1. Materials

Alginic acid sodium salt from brown algae (BioReagent, suitable for immobilization of microorganisms; 71238), calcium chloride (CaCl_2_; C1016), albumin from bovine serum (BSA; A2153), sodium citrate dihydrate (C_6_H_5_Na_3_O_7_∙2H_2_O; W302600), sodium chloride (NaCl; 746398), sodium hydroxide (NaOH; S5881), potassium hydrogen phthalate (C_8_H_5_KO_4_; P1088), barium chloride (BaCl_2_; B0750), sodium phosphate monobasic dihydrate (NaH_2_PO_4_∙2H_2_O; 71505) and sodium phosphate dibasic dehydrate (Na_2_HPO_4_∙2H_2_O; 71643) were obtained from Sigma-Aldrich (St. Louis, MO, USA). Hydrochloric acid (1N) was obtained from Merck KGaA (Darmstadt, Germany). For protein quantification, a bicinchoninic acid assay (BCA) kit (Visual Protein, Energenesis Biomedical Co., LTD., Taiwan) was used.

### 2.2. Preliminary Study for Formation and Characterization of Microparticles

Alginate microparticles containing the antigen were produced as reported in our previous study [17] using an AAJ system.

Preliminary studies were carried out to evaluate the effect of different processing conditions on the formation of microparticles. Sodium alginate solutions (1, 2, 3% *w*/*v*) were prepared by mixing the alginate powder with distilled water (100 mL). Suspension was maintained under gentle agitation (2–3 h, 300 rpm, 25 °C) until complete hydration. A volume of 1 mL of antigen solution (3.5 mg/mL total protein, *P. salmonis* antigen AUS005 produced as reported by Pontigo et al. [36] was added to a previously prepared alginate solution (20 mL), which was kept in continuous agitation (300 rpm) for 15 min. The alginate solution containing the antigen was pushed using a syringe pump (NE 8000, New Era Pump Systems Inc., Farmingdale, NY, USA) into a hydropneumatic nozzle (XAPR 200A 303 SS, BETE Fog Nozzle, Inc., Greenfield, MA, USA) where the solution was dispersed in the form of a jet with the help of a compressed air stream. The droplets were dripped into a crosslinking solution (500 mL of CaCl_2_, 0.25 M) from a height (distance between jetting nozzle and CaCl_2_ solution) of 25 cm. The microparticles formed were kept for 30 min in the calcium chloride solution to ensure crosslinking.

The effect of changes in alginate concentration (1, 2 and 3% *w*/*v*) and pumping flow rate (200, 400 and 600 mL/h) on the morphology of the alginate-antigen microparticles was evaluated by optical microscopy (BX43, Olympus Corporation, Tokyo, Japan) at 60× magnification. Images were obtained using the QCapture Pro 7^TM^ software (QImaging, Surrey, BC, Canada). An air pressure of 1.5 bar was applied for this evaluation.

The effect of changes in the air pressure (0.5, 1, 1.5, 2 and 2.5 bar) or pumping flow rate (50, 100, 150 and 200 mL/h) on the particle size obtained were evaluated by dynamic light scattering in a Zetasizer (Nano-ZS90, Malvern Instruments, Worcestershire, UK). The measurements were performed on diluted (1:100 distilled water) samples. The pumping flow rate was set at 100 mL/h for the evaluation of the air pressure effect, and the air pressure was set at 1.5 bar for evaluation of the effect of the pumping flow rate. An alginate concentration of 3% *w*/*v* was used in these evaluations.

### 2.3. Optimization of Microparticle Encapsulation Efficiency

To optimize antigen encapsulation efficiency (%) of the prepared microparticles, an experimental Taguchi design was applied (Table 1). An orthogonal matrix L9 (3^4^) with four independent variables and three levels of work (L1, L2 and L3) was used, applying the criterion “bigger is better”: flow rate of alginate solution (variable A; L1: 50, L2:125, L3: 200 mL/h), alginate concentration (variable B; L1: 1, L2:2, L3:3% *w*/*v*), distance between jetting head and crosslinking solution (variable C; L1: 15, L2: 20, L3: 25 cm), and air pressure (variable D; L1:0.5, L2: 1.25, L3: 2 bar). The optimized theoretical equation (OTE) was determined by considering the average of the response with the greatest impact, identifying the most important variables and levels of work.

#### Determining Encapsulation Efficiency

Microparticles in the solution were centrifuged (U-320 R centrifuge, Boeco, Germany) at 4000 rpm for 10 min and the calcium chloride supernatant was removed. The obtained microparticles were rinsed with distilled water and centrifuged again. Protein content was determined by dissolving an exactly weighed amount (3 g) of microparticles in 3% *w*/*v* sodium citrate water solution (15 mL) for 2 h at room temperature with magnetic stirring, breaking down the structure entrapping the protein [37]. After centrifugation (5415 R Centrifuge, Eppendorf AG, Hamburg, Germany) at 10,000 rpm for 10 min, protein concentrations in the clear supernatant solutions were assayed using an absorbance reader (800TM TS, Biotek Instruments, Inc., Winooski, VT, USA) using the BCA method [38], with BSA as the standard.

Encapsulation efficiency was expressed as the percentage of encapsulated antigens in relation to the initial number of antigens used:

(1) $$EE\left(\%\right)=\frac{microencapsulated\;antigens}{total\;initial\;antigen\;amount}\times 100$$

### 2.4. Evaluating Stability of Encapsulated Antigens

The effect of different conditions (pH, temperature, salinity, fresh water, sea water, storage and simulated fish gastrointestinal conditions) on the microparticle stability was evaluated. Five grams of microparticles were dispersed into a 50 mL solution with the corresponding environmental condition and incubated for 24 h (except for storage and digestion studies). To determine protein content after the treatment, microparticles were processed as explained in the previous section. The remaining protein content (RPC%) was calculated as follows:

(2) $$RPC\;\left(\%\right)=\frac{Protein\;content\;after\;incubation}{Initial\;protein\;content}\times 100$$

Microparticles used in the stability assessment were produced in the optimized conditions defined above. BSA was used as a model protein to evaluate the effect of different conditions on the stability of protein-loaded alginate microparticles. Polymeric solutions (60 mL) had a concentration of 1mg/mL BSA protein.

#### 2.4.1. Evaluating the Effect of pH, Temperature and Salinity

To evaluate the effect of pH, the microparticles were dispersed in solutions with pH from 2 to 10. Solution pH was manually adjusted at two-unit intervals with the aid of a pHmeter (HI-2002 Edge, Hanna Instruments, Woonsocket, RI, USA) using 0.1 M HCl or 0.5 M NaOH solutions. In the case of temperature, the effect was evaluated in solution (distilled water) and as humid microparticles (recovered after centrifugation). The samples were exposed to temperatures from 5 to 30 °C in steps of 5 °C using a refrigerated incubator (Bioref-19 L, PiTecnologia S.A, Santiago, Chile). The influence of ionic strength on the microparticle stability was determined by dispersing the microparticles in solutions with a salt concentration between 0 and 40 g/L NaCl.

#### 2.4.2. Microparticle Stability in Fresh Water and Sea Water

With the aim of simulating the natural conditions that the microparticle will encounter when it is supplied to the fish, the stability of the microparticles in fresh water and sea water were evaluated.

Fresh water and sea water were supplied by the Cargill Innovation Centre (Colaco, Chile), where water from a deep well or the sea from Colaco bay are treated, first removing solids through a sand filter and then applying UV light (350 µJ/cm^2^) to eliminate microorganisms. Fresh water had a pH of 8.2 and a salinity of 2 PSU; sea water had a pH of 7.4 and a salinity of 35 PSU.

Five grams of microparticles were dispersed in the solution (50 mL) and kept in solution at 10 °C (Bioref-19 L, PiTecnologia S.A, Santiago, Chile) for 24 h. Then, the microparticles were recovered and the protein content was determined as explained previously.

#### 2.4.3. Storage Stability

Stability of the microencapsulated antigens under different storage conditions was evaluated. Microparticles were produced under optimized conditions and stored for 30 days as: (i) recovered microparticles at 4 °C; (ii) recovered microparticles at 25 °C; (iii) microparticles in calcium chloride solution at 4 °C, and (iv) microparticles in calcium chloride solution at 25 °C. After the storage period, protein content was determined as explained previously.

#### 2.4.4. Simulated Fish Gastrointestinal Conditions

To simulate fish gastrointestinal conditions, microparticles were evaluated in an in vitro two-stage assay as previously described [29]. First, microparticles were dispersed into an acidic dissolution medium (KPH/HCl, pH 3.0) and kept under magnetic stirring (200 rpm) to simulate gastric conditions. After 15 min, microparticles were recovered by centrifugation and submerged into an alkaline medium (phosphate buffer pH 8.0, 200 rpm) for 60 min to simulate the intestinal conditions in Atlantic salmon. A temperature of 10 °C was applied to replicate as fully as possible the natural conditions under which salmon habitually live. The remaining protein content was determined as explained before by taking samples of 5 g at the end of the acidic and the alkaline phases.

Buffer solutions were prepared as follows: (a) a potassium hydrogen phthalate/hydrochloric acid (KPH/HCl, pH 3.0) buffer was prepared by combining 0.4 M KPH (500 mL) with 0.4 M HCl (223 mL). The resulting buffer solution was adjusted to pH 3.0 before replenishing with distilled water to give a total volume of 1000 mL; (b) phosphate buffer (pH 8.0) was created by combining 0.4 M monosodium phosphate dihydrate solution (26.5 mL) with 0.4 M disodium phosphate dihydrate solution (473.5 mL). The resulting buffer solution was adjusted to pH 8.0 and then diluted with distilled water to a final volume of 1000 mL.

### 2.5. Assessment of Microparticle Incorporation in Fish Feed Pellets

The experimental oral vaccine in feed was generated as in our previous study [17] using a vacuum coater (F-6 RVC, Forberg International AS, Oslo, Norway). Briefly, the obtained microparticles in the solution (128 g) were mixed with fish oil (672 g) using a digital Ultra TURRAX (T50, IKA^®^-Werke GmbH & CO., Staufen, Germany) at 6000 rpm for 2 min. Then, 4.2 kg of base pellet (Micro 50, EWOS, Coronel, Chile) and the microparticles mixed with fish oil (800 g) were incorporated into the mixing chamber of the vacuum coater. The ingredients were mixed and the vacuum coating process was conducted.

To detect the microparticles and observe their distribution inside the coated pellet, one fish feed pellet (height: 4 mm, diameter: 2 mm) was scanned by high-resolution micro-computed tomography (Skyscan 1272, Bruker, Kontich, Belgium) operating at 45 kV of source voltage and a constant source current of 222 μA. For this study, the alginate microparticles were produced using barium chloride (0.25 M) as a cross-linking solution instead of calcium chloride, since barium is commonly used as a contrast agent in such analyses [35]. Two-dimensional visualization of a pellet cut along its central axis was obtained through Data Viewer (Bruker, Kontich, Belgium), the software associated with the equipment. Comparative images were taken adjusting the color/data range with the image control of the software set at a grey scale. A tridimensional image of the pellet was obtained through the Bruker CTvox (Version 3.1.1) software (Bruker, Kontich, Belgium); microparticle visualization was obtained by adjusting the opacity and the luminance channels in the transfer editor function of the program, highlighting the structures of maximum intensity.

### 2.6. Statistical Analysis

Results were expressed as mean values and standard deviations calculated from measurements of replicate samples. Data were checked for normality (Shapiro-wilk test) and homoscedasticity (Fligner-Killeen test). Particle size and RPC were analyzed using a non-parametric Kruskal–Wallis test, followed by post hoc test using the Holm *p*-value adjustment method to determine significant differences at *p* ≤ 0.05. All statistical analyses were performed using R (version 4.0.2) and the R package agricolae (version 1.3-5).

## 3. Results

### 3.1. Preliminary Study for the Formation and Characterization of Microparticles

The AAJ method was used for the dispersion of the alginate solution containing the *Piscirickettsia salmonis* antigen. Preliminary studies were carried out to determine the working ranges in the AAJ that would allow the formation of round microparticles with a particle size small enough (<100 µm) to be incorporated into the porous matrix of the fish feed pellet [29].

**Figure 1:**
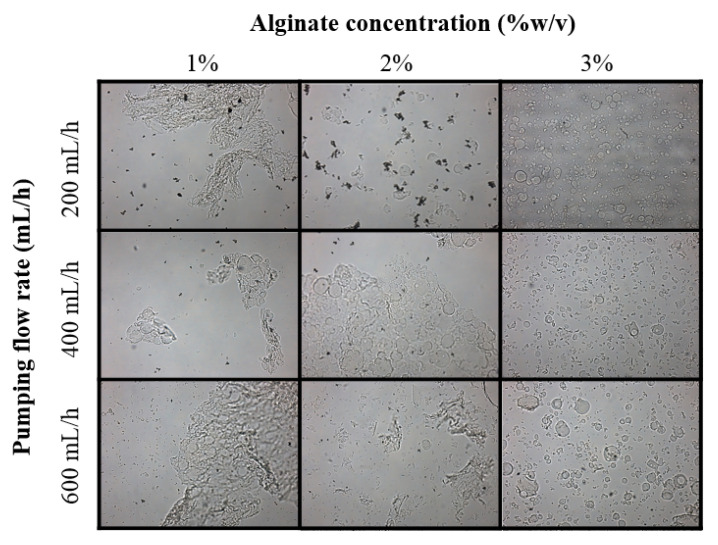
Optical microscopy (60×) of microparticles obtained under different processing conditions: alginate concentration (1, 2 and 3% *w*/*v*) and pumping flow rate (200, 400 and 600 mL/h). Other working conditions of the AAJ: CaCl_2_ concentration: 0.25 M; distance: 25 cm; air pressure: 1.5 bar.

The effects of alginate concentration of the polymeric solution and the pumping flow rate of the AAJ system on the microparticle morphology were evaluated. It was observed that with a lower concentration of alginate, aggregations and particles in the form of flakes were obtained; nevertheless, using higher concentrations of alginate and lower flow rates, round microparticles without aggregations could be obtained (Figure 1).

**Figure 2:**
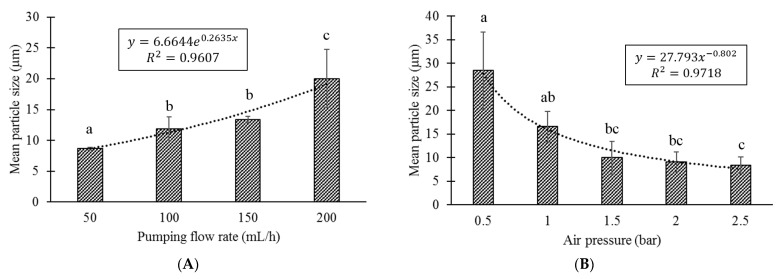
The influence of pumping flow rate (**A**) or air pressure (**B**) of the aerodynamically assisted jetting (AAJ) system on the mean particle size of the microparticles obtained. Working conditions of the AAJ were: alginate concentration: 3% *w*/*v*; CaCl_2_ concentration: 0.25 M; distance: 25 cm; flow rate: 100 mL/h (**A**); air pressure: 1.5 bar (**B**). Different lowercase letters indicate significant differences (*p* ≤ 0.05) of the mean particle size among the different pumping flow rates or air pressures evaluated.

The influence of pumping flow rate or air pressure of the AAJ system on the mean particle size of the microparticles obtained was also evaluated. A significant increase (*p*-value: 0.02245) in particle size (PS) was observed when the flow rate (FR) was increased from 50 to 200 mL/h, described by the following equation: $PS=6.6644\times e^{0.2635\times \left[FR\right]};R^{2}=0.9607$. In terms of pressure, the particle size was significantly reduced (*p*-value: 0.03044) as the air pressure (AP) was increased, as described by the power law model ($PS=27.793\times {\left[AP\right]}^{-0.802};R^{2}=0.9718$). The mean particle size was less than 10 microns when pressure higher than 1.5 bar was applied using a flow rate of 100 mL/h (Figure 2B).

### 3.2. Optimization of Microparticle Encapsulation Efficiency

Once working ranges that allow microparticle formation had been determined, the antigen encapsulation efficiency was optimized using the Taguchi method with an orthogonal array L9(3^4^). The effect of four variables were evaluated: flow rate, alginate concentration, distance and pressure. Table 2 shows EE% results and the microparticle mass obtained under the different conditions of the experimental design. After evaluating each design point, EE% values varied between 80.31 and 95.72%, and microparticle mass, between 5.22 and 22.56 g, after dispersing 60 mL of polymeric solution.

**Figure 3:**
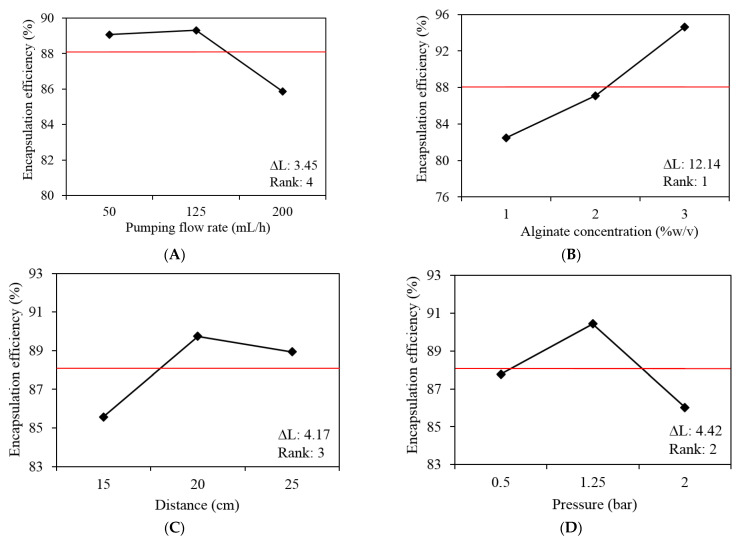
The effect of each variable working level on the antigen encapsulation efficiency. (**A**) Pumping flow rate, (**B**) Alginate concentration, (**C**) Distance and (**D**) Pressure. The red line represents the total average responses of experimental runs, ∆L corresponds to the size of the effect by taking the difference between the highest and lowest characteristic average for a factor, Rank represents the order of the variables from those with the largest effect to the least.

Figure 3 shows the slope of the incline between the points as a response to EE%. The greater the difference between the levels for a variable, the higher the magnitude change of the response. The alginate variable had a greater influence on the EE%, with an average difference of 12.14 units between responses, followed by pressure, with a difference of 4.42 units, distance with a difference of 4.17 units, and finally, flow rate with a difference of 3.45 units between working levels.

According to the results obtained from the experimental Taguchi design (Figure 3), medium flow rate (125 mL/h), higher alginate concentration (3% *w*/*v*), medium distance (20 cm) and medium pressure (1.25 bar) resulted in a high encapsulation efficiency. The optimized theoretical equation (OTE) was determined by considering the average of the response with the greatest impact, identifying the most important variables and levels of work:

OTE=T+[(Flow rate, L2)−T]+[(Alginate%,L2)−T]+[(Distance,L2)−T]+[(Pressure,L2)−T]

where *T* is equal to 88.08%, which is the total average responses of experimental runs, and *L* corresponds to the working level of the equation. The optimized theoretical value determined by the OTE was 99.89%. To test the theoretical equation, microparticles were prepared using the optimized levels. The EE% obtained using the optimized levels was 97.92% and microparticles had a mean diameter of 21.4 μm.

### 3.3. Stability of Encapsulated Antigens

#### 3.3.1. Effect of pH, Temperature and Salinity

The effects of different environmental conditions (pH, temperature and salinity) on the microparticle stability were evaluated.

**Figure 4:**
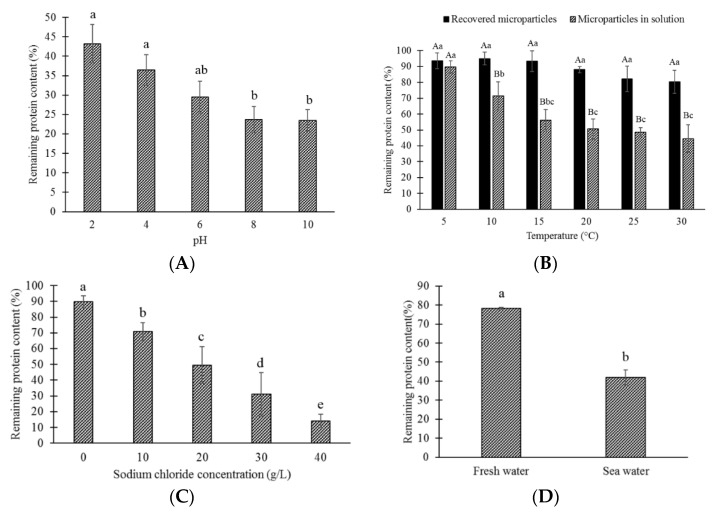
The effect of different environmental conditions on microparticle stability and protein release. (**A**) Effect of pH; (**B**) Effect of temperature; (**C**) Effect of salinity; (**D**) Effect of type of water. Different lowercase letters indicate significant differences (*p* ≤ 0.05) of the remaining protein content (%) among the different points of evaluation within the same type of sample. Different capital letters in Figure 4B indicate significant differences (*p* ≤ 0.05) of the remaining protein content among the different types of samples (recovered microparticles, microparticles in solution) within the same temperature.

Microparticles were exposed to solutions with different pH (2–10) for 24 h and the remaining antigen content was determined (Figure 4A). Changes in the pH of the dispersion medium had a strong effect on the stability of the microparticle. More than 50% of the antigen was lost in all samples. The remaining protein content (RPC) decreased significantly (*p*-value: 0.02222) with increasing pH ($RPC=-13.2\times \ln\left(pH\right)+43.89;R^{2}=0.9738$). The lowest protein content was observed between pH 8 and 10, where only 23% protein was retained.

The effect of temperature on the stability of the microparticles was evaluated by applying temperatures between 5 and 30 °C (Figure 4B). In this study, the microparticles were evaluated as dispersed in the solution and as recovered humid microparticles. A decrease in the protein content of the microparticles was observed as the temperature increased, represented by the equation $RPC=90.384\times T^{-0.4};R^{2}=0.9875$, for microparticles in solution and by: $RPC=-3.1605\times T+99.793;R^{2}=0.8757$, for recovered microparticles. There were no significant differences (*p*-value: 0.06799), however, in the RPC of the recovered microparticles after being exposed to different temperatures. The release was significantly higher (*p*-value: 0.02011) when the microparticles remained in solution than when they were recovered and kept as humid microparticles. For example, at 30 °C the remaining protein content was only 43.48% for microparticles in solution, while the recovered microparticles kept 80.27% of the protein.

The influence of ionic strength was evaluated by dispersing the microparticles in solutions with a sodium chloride concentration from 0 to 40 g/L. When the microparticles were exposed to the solutions, the remaining protein content decreased linearly ($RPC=-19.114\times \left[NaCl\right]+108.4;R^{2}=0.9985$) as the sodium chloride concentration increased (Figure 4C), generating a significant change (*p*-value: 0.01019) in the protein content of the microparticle. Almost all protein content was released when the sodium chloride concentration was 40 g/L, and only 14% was retained after 24 h.

Furthermore, microparticle stability on fresh water or sea water was assessed (Figure 4D). The remaining protein content was significantly higher (*p*-value: 0.003326) for microparticles kept in fresh water. A retention of 78.25% of the protein content was obtained in fresh water, while in seawater only 42% was retained, meaning that 58% of the protein content of the microencapsulate was lost in seawater.

#### 3.3.2. Storage Stability

**Figure 5:**
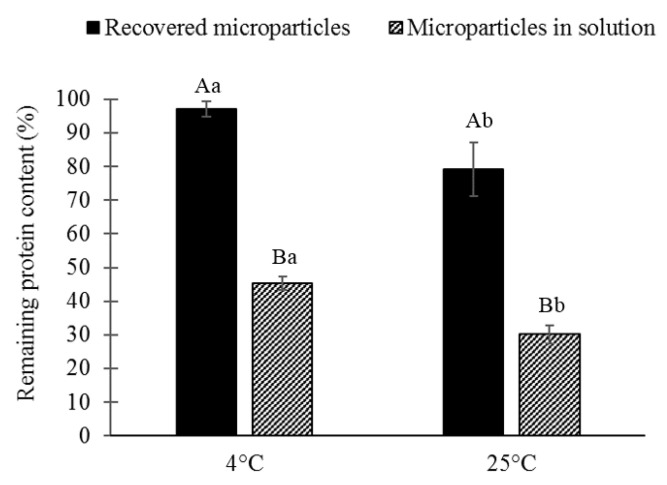
The remaining protein content after 30 days of storage. Microparticles were stored as recovered humid microparticles or in solution at 4 °C or 25 °C. Different lowercase letters indicate significant differences (*p* ≤ 0.05) of the remaining protein content (%) among the different temperatures within the same type of sample. Different capital letters indicate significant differences (*p* ≤ 0.05) of the remaining protein content among the different types of samples (recovered microparticles, microparticles in solution) at the same temperature.

The conditions of storage showed a high influence on the microparticle stability (Figure 5).

High losses of protein content were observed in microparticles kept in the solution, and the RPC was 45.35% and 30.18% for microparticles stored at 4 °C and 25 °C, respectively. The RPC was significantly higher (*p*-value: 0.01556) for samples stored as humid microparticles (recovered after centrifugation), 97.13% and 79.12% for microparticles stored at 4 °C and 25 °C, respectively. Storing the microparticles in the solution generated losses of ~50% more than when they were recovered. Furthermore, storing the microparticles at room temperature resulted in losses of ~15% more than when stored under refrigeration conditions. The highest protein retention (97.13%) was obtained by storing the microparticles without solution at a refrigeration temperature (4 °C).

#### 3.3.3. Stability under Simulated Fish Gastrointestinal Conditions

**Figure 6:**
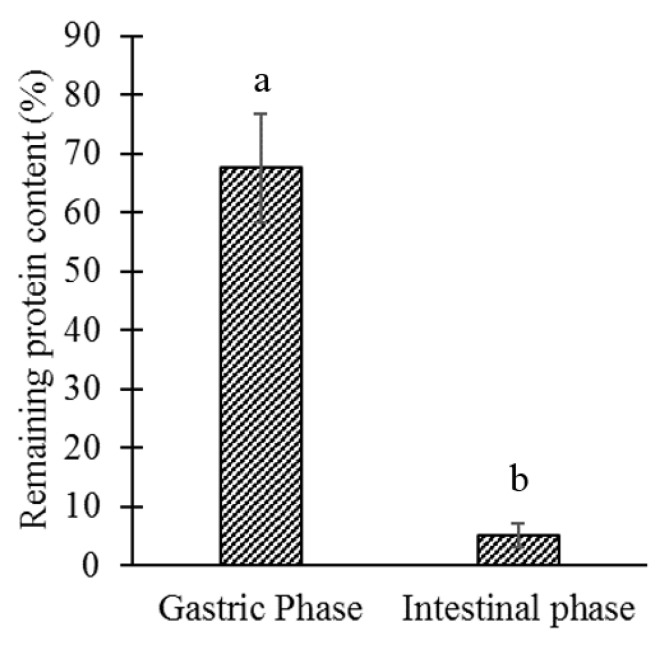
The remaining protein content after exposing microparticles to simulated fish gastrointestinal conditions. Microparticles were evaluated in an in vitro two-stage assay: (i) Gastric phase: acidic dissolution medium (KPH/HCl, pH 3.0) for 15 min. (ii) Intestinal phase: alkaline medium (phosphate buffer pH 8.0) for 60 min. Different lowercase letters indicate significant differences (*p* ≤ 0.05) of the remaining protein content (%) among the different phases of digestion.

Finally, microparticles were evaluated in an in vitro two-stage assay, simulating the temperature and pH conditions that would be found in the gastrointestinal tract of salmon (Figure 6).

In the gastric phase, the microparticles were exposed to an acidic pH for 15 min, during which time 32.46% of the protein content was released. In the intestinal phase, microparticles were exposed to solution with pH 8 at 10 °C for 60 min. The remaining protein content was significantly (*p*-value: 0.00339) lower than in the gastric phase: almost all protein content was released, leaving only 5.12% in the microparticles; consequently, 62.42% of the protein content is released at the intestinal level and accessible for absorption.

### 3.4. Assessment of Microparticle Incorporation in Fish Feed Pellets

Microparticle incorporation in fish feed pellets was evaluated through a micro-CT scanning analysis. For better visualization, microparticles were produced using barium as a cross-linking agent. Barium alginate microparticles had an average particle size of 40.53 ± 19.31 µm, an amount of 18.07 g of microparticles in solution were added to the oil mix and then incorporated into the pellet by vacuum infusion.

**Figure 7:**
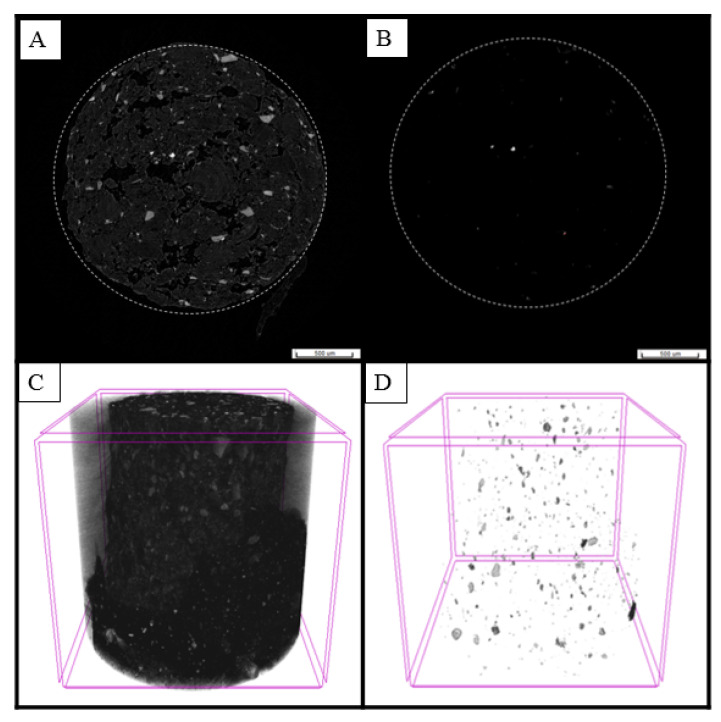
Micro-CT scan images of a pellet infused with barium-alginate microparticles. (**A**) 2D visualization; (**B**) 2D visualization of infused microparticles; (**C**) 3D visualization; (**D**) 3D visualization of infused microparticles. Measurement scale in the corner of images A and B indicates 500 µm.

2D visualization of the fish feed pellet infused with microparticles was obtained using the Data Viewer software (Figure 7A); the program delivers cross sections along the Z axis of the pellet, and 1254 images were obtained. A representative picture was taken at the position 827 of the Z axis and comparative images were taken with the image control set at a grey scale. The original picture was taken at a Color/Data range of 0/255 and a microparticle visualization picture (Figure 7B) was obtained at a Color/Data range of 135/255, where it is possible to observe the structures with higher intensity and particles with a size and shape similar to the barium alginate microparticles, confirming microparticle incorporation.

In addition, a 3D image of the pellet was obtained through Bruker CTVox program (Figure 7C). Adjusting the opacity and luminance channels in the Transfer Function Editor of the program makes it possible to see the structures with higher intensity, while a similar picture could be obtained applying the “maximum intensity projection” mode. The resulting picture is observed in Figure 7D, where it is possible to observe the alginate barium microparticles inside the pellet and their uniform distribution. Particles with sizes between 20 and 40 microns inside the pellet were detected, which should correspond to the microparticles infused by vacuum coating on the pellet.

## 4. Discussion

One of the main issues in fish vaccine development is improving current oral vaccination strategies [2]. In a previous study, the possibility of developing a fish oral vaccine by incorporating a microencapsulated *P. salmonis* antigen in fish feed has been confirmed, effectively enhancing the immune response of Atlantic salmon [17]. Consequently, in this study we sought to determine the best conditions for microparticle formation, optimize encapsulation efficiency and evaluate conditions that might affect microparticle stability improving our current vaccination strategy.

Alginate microparticles containing the *P. salmonis* antigens were effectively produced using the AAJ system. This technology has been tested effectively for microencapsulation of biological materials including cells and whole organisms [39]. Furthermore, in recent years this technology has been used for the development of fish oral vaccines for IPNV [13,29] and SRS [17]. Preliminarily, conditions for the formation of soft round microparticles were evaluated. It was observed that high pumping flow rates were not effective for the formation of droplets, leading to the formation of flake-shaped particles and agglomerations. This effect has been previously described for this type of device, where adjusting flow rate and pressure conditions, threads, droplets and scaffolds can be formed [40]. Higher alginate concentrations produced round microparticles regardless of the pumping flow rate used; studies suggest that a higher proportion of wall material will allow the formation of a stronger encapsulating matrix and a better cross-linking on the bead surface [28].

A small particle size was also sought, considering these microparticles must be incorporated in the pores of fish feed pellets with pore sizes in a range between 10 and 500 microns [29]. Additionally, a small particle size allows better bioactive absorption in the intestine [41]. Both pumping flow rate and air pressure influenced the mean particle size obtained; particle sizes less than 20 microns were obtained with pumping flow rates less than 200 mL/h and pressures greater than 1 bar (under the conditions used in these evaluations). A previous study reported that the average droplet sizes observed in the jet formed by an AAJ device were between 55 and 87 μm [40]; furthermore, protein encapsulation with alginate has been reported in the literature, obtaining microparticles with mean particle sizes between 3 μm [37] and 200 μm [42]. Therefore, microparticles obtained in our study were within expected size ranges.

Subsequently, optimization of encapsulation efficiency was carried out through the Taguchi method. An experimental antigen encapsulation efficiency of 97.92% was achieved using a flow rate of 125 mL/h, an alginate concentration of 3% *w*/*v*, a distance of 20 cm and air pressure of 1.25 bar. This result coincides with that reported in a previous study using the same technology for the encapsulation of IPNV antigens where encapsulation efficiencies between 79 and 97% were obtained depending on the compound used for cross-linking [29,31]. The Taguchi method identified the process variables that most affected encapsulation efficiency with the minimum number of tests, confirming that the combination between the control variables and working levels were sufficient to increase EE%.

Among the parameters evaluated, pumping flow rate had the least effect on the encapsulation efficiency. As illustrated in Figure 2A, there is no significant change when the pumping flow rate varies between 50 and 125 mL/h. Although a lower flow rate generates smaller microparticles, this speed may not be enough to effectively disperse microparticles from the nozzle; in addition, for productivity purposes a higher flow rate is preferable. Alginate concentration showed great influence on the antigen encapsulation efficiency. This effect has been reported previously, where a higher concentration of alginate allows better crosslinking, creating an encapsulation matrix that avoids bioactive leakage [28,43]. Finally, the distance between the jetting head and the cross-linking solution and the air pressure of the system are two operational variables that could affect the adequate formation of the microparticle, consequently affecting the encapsulation efficiency. In terms of distance, with a very high distance the microparticles would not reach the CaCl_2_ solution, adhering to the collector vessel or land elsewhere than onto the surface of the cross-linking solution. On the other hand, a very small distance could generate a backflow that would block the exit of the nozzle. In terms of air pressure, a very low pressure would not be effective for the dispersion of the microparticles from the nozzle; however, a very high pressure could also generate backflow effects, reducing the productivity. One of the problems associated with this technology were the losses produced by dispersing the polymeric solution, where, after dispersing 60 mL of solution, a maximum of 23 g of microparticles were obtained. A portion of these losses could be associated with the volatilization of small particles, while another smaller portion of microparticles is lost by adhering to the collecting vessel.

After a high EE% was achieved, the effect of different environmental conditions (pH, temperature and salinity) that the microparticle might encounter in the process of producing the oral vaccine, in its storage, supply or in being digested by the fish, affecting microparticle stability, were evaluated. Changes in the pH of the dispersion had a strong effect on the microparticle stability, which can be attributed to differences in electrostatic interactions between alginate and proteins. At acidic pH, the protein and alginate have opposite charges and are strongly attracted to each other, and therefore the protein is better retained within the alginate microparticles. At a higher pH, protein and alginate have similar charges, which generate an electrostatic repulsion; consequently, the protein tends to leach out from the microparticles [44]. The pH dependence on the protein release could also be the effect of changes in the structure of alginate capsules under different conditions. It has been reported that alginate microparticles tend to shrink at low pH due to the loss of the negative charge on the alginate molecules when the carboxyl groups become protonated [45]. On the other hand, the microparticles tend to swell when they are dispersed in a solution with a higher pH due to the fact that alginate molecules become highly charged and repel each other [46]. In the same way, it was observed that temperature could have an effect on the stability of the microparticles, especially when they are kept in solution. Studies suggest that alginate beads are generally thermostable between 0 and 100 °C; however, exposure to temperature for prolonged periods can affect the polymeric structure of the microparticle, and therefore lead to a more rapid diffusion of the bioactive [47], explaining the reduction of RPC with increasing temperature. The highest stability in microparticles evaluated without solution can be produced by the significant reduction in porosity of alginate particles when partially dried [48]. The effect of salinity was evaluated, dispersing the microparticles in solutions with a sodium chloride concentration from 0 to 40 g/L. Linear reduction of the RPC was observed as the salinity increased. The increase in protein release could be a side effect of the high presence of ions generated by the dissociation of sodium chloride in solution. As with the pH, the presence of ions in the dispersion medium could affect the structure and stability of the microparticle; in this case, the positively charged counter ions (Na+) from the salt could generate a screening effect on the negative charges of the microparticles [49].

As expected, this effect was also observed by evaluating the stability of microparticles in freshwater and seawater, where 36.25% more was retained in fresh water. Fresh water usually has a salt concentration lower than 5 g/L and sea water has a high salinity that may vary between 33 and 37 g/L [50]. In general, vaccinations to prevent infections are carried out in fresh water before smoltification [51]; however, given the recurrent outbreaks after immunization [52], it is important to evaluate the use of this vaccine as an oral booster when the fish are already in seawater. Given these results, slight protein losses should be expected when the oral vaccine is supplied in seawater, although they probably will not be as high as the 58% obtained in the study, the microparticles will be in contact with water for a much shorter period since fish will consume the food almost immediately after being supplied. A previous study evaluated the stability of alginate microparticles in sea water, determining that the losses depended on the protein load of the microparticles and the exposure time, so that the losses could be minimized by adjusting the protein load in the microparticles, being less than 2% for up to 18 h of exposure [53].

On the other hand, the use of other additional polymers that can prevent or slow down the release of the antigen against environmental conditions that affect the structure of alginate microparticles could be considered. A combination of calcium alginate with other ingredients such as chitosan could lead to a new way of controlling the delivery of bioactive compounds. The positively charged chitosan provide a semi-permeable membrane with the negatively charged alginate resulting in a smoother capsule which is less permeable to water soluble molecules [54]. The preservative effect of chitosan might be due to the strong binding of chitosan to alginate spheres via electrostatic interactions providing a strong membrane on the surface of the spheres, decreasing the leakage of the encapsulated ingredients [55].

Regarding long- term storage, as in the study reported by Vega–Carranza et al. [56] it was determined that the best storage temperature was 4 °C. The results of this study are consistent with those obtained in the study of the effect of temperature, confirming how a higher temperature can affect the polymeric structure of the microparticle, allowing a greater protein diffusion. Likewise, it is confirmed that keeping the microparticles partially dry can considerably reduce the porosity of the particle, reducing the release of proteins [48]. It was determined that the microparticles should be stored as dry microparticles in refrigerated conditions to reduce protein release.

Furthermore, the effect of conditions (pH and temperature) to partially simulate the fish gastrointestinal environment were evaluated. The objective of this study was to determine if microencapsulation can protect the antigen from the acidic medium of the stomach and deliver the bioactive at the intestinal level. As expected, protein release was higher in the intestinal phase, this effect mainly being caused by the pH, where electrostatic repulsion is generated between the protein and the polymer matrix, generating greater leaching. At this pH, the microparticles tend to swell and eventually become more soluble and viscous, losing all their structural integrity [57]. Almost all protein content was released at intestinal level, indicating that this is a good delivery system that can partially protect the antigen at the gastric phase and release the antigens for their absorption.

On the other hand, it should be considered that the encapsulated antigens will later be incorporated into the feed to generate the oral vaccine. After being ingested, the feed that enters the stomach begins to disintegrate by motility and peristalsis together with the presence of HCl and pepsin that start pre-digestion of proteins [58], in this process the microparticles will be liberated and exposed to the gastric environment; therefore, slight changes in the release profiles could be expected, given that our simulation model was executed directly on the microparticles and lacks digestive enzymes.

Finally, microparticle incorporation in fish feed pellets was evaluated through a micro-CT scanning analysis. The vacuum coating method was used for the incorporation of antigens against IPNV [14], *Streptoccoccus iniae* [59] and *P. salmonis* [16,17] with effective results. In this study, the antigen was microencapsulated and then incorporated in the fish feed, alginate-antigen microparticles were produced using a barium salt (BaCl_2_) as cross-linking instead of the calcium chloride (CaCl_2_) commonly used, given that barium is generally used as a contrast in computed tomography performed in human medicine for better visualization [60].

The fish feed pellets have a highly irregular pore shape, and pore sizes are in the range of 10 to 500 µm, where the majority have a size close to 100 µm, and there is another large proportion with sizes close to 400 µm [29]. Furthermore, Draganovic et al. [61] reported that most pore sizes in the fish feed pellets (davg = 8.7 mm) were below 330 μm; therefore, we expected these microparticles to be incorporated in the pellet, as reported in a previous study, where evidence of the incorporation of the microencapsulate was obtained by detecting calcium-associated particles with a similar shape and size by scanning electron microscopy and energy dispersive X-ray spectroscopy (SEM-EDS) [17]. Micro-CT scanning enables us to confirm the incorporation of microparticles in the pellet and to visualize their distribution within it. It should be noted that barium alginate microparticles had a slightly bigger mean particle size (40.53 µm) than the calcium alginate microparticles (21.4 µm); therefore, a greater incorporation of the calcium alginate microparticles could be expected. Although this study cannot verify it, it is expected that microparticles with a mean particle size greater than 100 µm will be trapped on the surface of the pellet exposed to external conditions and also prevent the incorporation of smaller particles by blocking the pore. In conclusion, smaller particles are preferred to enable a high degree of inclusion in the fish feed pellets and uptake by the distal intestine. Additional research could be performed to identify the largest suitable particle size in relation to a given pore size of feed pellet.

## 5. Conclusions

These results show that the AAJ system is effective at encapsulating *P. salmonis* antigens and generating microparticles with diameters close to 20 microns. The Taguchi method made it possible to determine which of the process variables most affected product quality with the minimum number of assays, achieving an encapsulation efficiency of 97.92% using the optimized conditions. Environmental conditions such as pH, salinity and temperature can generate microparticle instability, triggering protein release; therefore, these conditions should be avoided to prevent antigen degradation or release, otherwise these effects should be considered when calculating the final dose of the vaccine. Additionally, it was determined that this oral delivery system would successfully release the antigen at intestinal level after evaluation under simulated fish gastrointestinal conditions. Finally, evidence of the microparticle incorporation in fish feed by vacuum coating was found by micro-CT scanning, confirming that this is a good system to produce a fish oral vaccine.

## Figures and Tables

**Table 1:** Taguchi design for optimization of antigen microencapsulation efficiency.

	Coded Variables				Non-Coded Variables			
Design Point	A	B	C	D	Flow Rate (mL/h)	Alginate (% *w*/*v*)	Distance (cm)	Pressure (bar)
1	1	1	1	1	50	1	15	0.5
2	1	2	2	2	50	2	20	1.25
3	1	3	3	3	50	3	25	2
4	2	1	2	3	125	1	20	2
5	2	2	3	1	125	2	25	0.5
6	2	3	1	2	125	3	15	1.25
7	3	1	3	2	200	1	25	1.25
8	3	2	1	3	200	2	15	2
9	3	3	2	1	200	3	20	0.5

**Table 2:** Antigen encapsulation efficiency using the orthogonal matrix L9(3^4^).

Design Point	Flow Rate (mL/h)	Alginate (% *w*/*v*)	Distance (cm)	Pressure (bar)	Encapsulation Efficiency (EE%)	Productivity ^1^ (grams)
1	50	1	15	0.5	80.66 ± 4.85	6.00 ± 0.62
2	50	2	20	1.25	92.11 ± 1.64	9.96 ± 0.57
3	50	3	25	2	94.41 ± 2.77	11.64 ± 0.16
4	125	1	20	2	83.33 ± 2.53	7.44 ± 0.76
5	125	2	25	0.5	88.89 ± 4.41	8.64 ± 0.80
6	125	3	15	1.25	95.72 ± 0.47	13.92 ± 0.56
7	200	1	25	1.25	83.50 ± 0.55	5.28 ± 0.43
8	200	2	15	2	80.31 ± 8.86	8.04 ± 0.58
9	200	3	20	0.5	93.77 ± 3.39	22.56 ± 0.77

^1^ Mass of microparticles produced after dispersing 60 mL of polymer solution.

## Data Availability

The data presented in this study are available on request from the corresponding author.
